# Supplementary material for: Scaling of Yu-Shiba-Rusinov energies in the weak-coupling Kondo regime
Source: Nat Commun. 2017 Dec 8;8:2016. doi: 10.1038/s41467-017-02277-7 (PMC5722882; doi:10.1038/s41467-017-02277-7)
Supplement: Supplementary file 1 — Supplementary Information [file 41467_2017_2277_MOESM1_ESM.pdf]

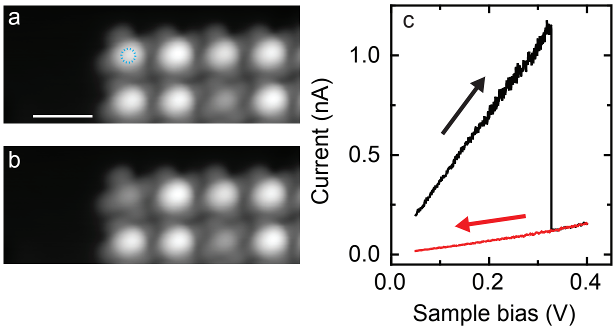

Supplementary Figure 1. Controlled NH_3 ligand desorption. (a,b) Topography of a mixed island of MnPc–NH_3 and MnPc before (a) and after (b) controlled desorption of an NH_3 ligand. Setpoint: 50 mV; 200 pA. Scale bar is 2.0 nm. (c) $I-V$ characteristic recorded on the position indicated with a blue circle in (a). The sudden drop in the current in the forward scan (black line) indicates the desorption of NH_3. The difference in conductance in the backward scan (red line) then proves the structural modification of the junction.

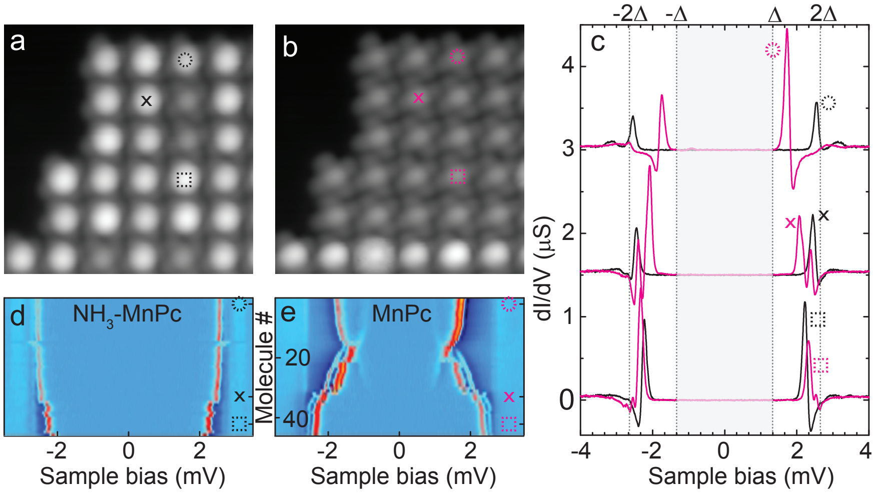

Supplementary Figure 2. Comparison of MnPc and MnPc–NH_3. (a,b) Topography of a molecular monolayer island of MnPc–NH_3 before (a) and after (b) voltage-induced desorption of some NH_3 ligands. Setpoint: 50 mV; 200 pA. (c) Three dI/dV excitation spectra of MnPc–NH_3 (black) as indicated in (a), and of the same molecules after NH_3 desorption (pink). (d) 2D color plot of dI/dV excitation spectra of 44 MnPc–NH_3 molecules ordered according to the order in (e). (e) 2D color plot of dI/dV excitation spectra of MnPc molecules (same as in (d), but after NH_3 desorption). Spectra are ordered according to the energy of the YSR resonance. Spectra acquired with a superconducting Pb tip. Setpoint: 5 mV, 200 pA; lock-in parameters: 912 Hz, 15 μV_{rms}.

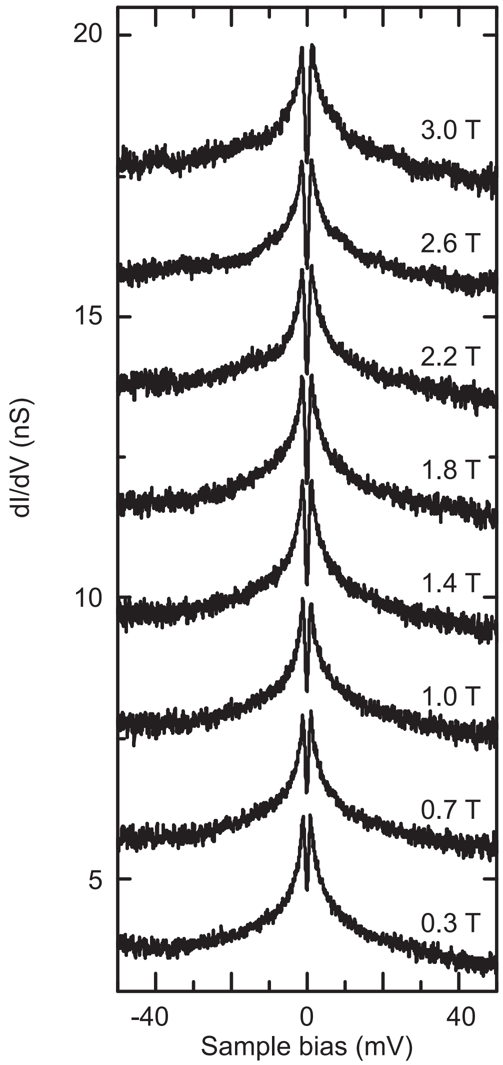

Supplementary Figure 3. B-field evolution of the zero-energy resonance. dI/dV spectra of MnPc–NH_3 at 1.1 K acquired with a normal metal Au tip. Setpoint: $U = 50\,\mathrm{mV}$, $I = 200\,\mathrm{pA}$; $U_{\mathrm{mod}} = 500\,\mu\mathrm{V}_{rms}$. Same molecule as in Fig. 3 of the main text.

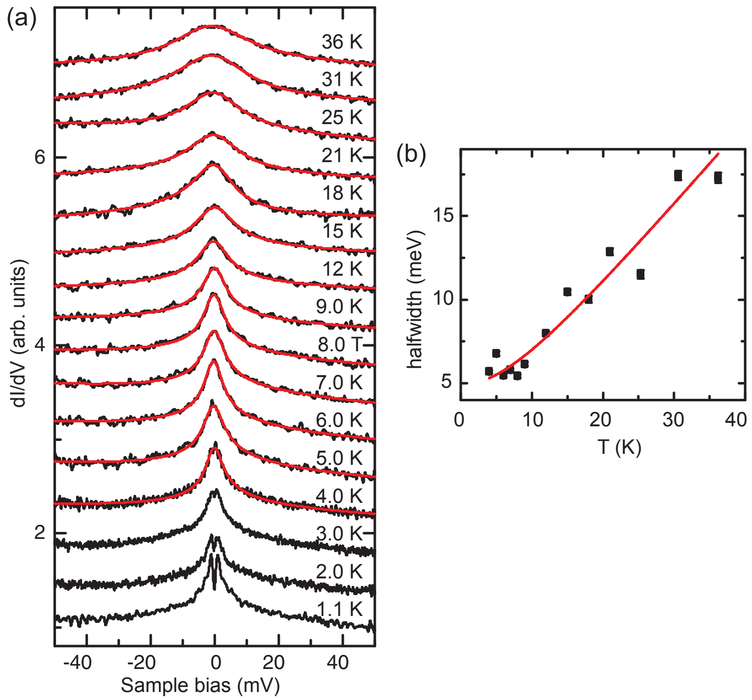

Supplementary Figure 4. Temperature-dependent zero bias resonance. (a) Temperature-dependent dI/dV spectra (same data as in Fig. 3a of the main manuscript) with a Fano-Frota fit, which accounts for temperature broadening (50 mV, 200 pA, 500 μV_{rms}). (b) Half width of the resonance as extracted from the Fano-Frota fit in (a) as a function of temperature. The red line is a fit describing the temperature evolution in the strong-coupling Kondo regime as described in the text. Fit parameters: $T_K = (57 \pm 5)\,K$; $\alpha = (3.7 \pm 0.2)\,\pi$.

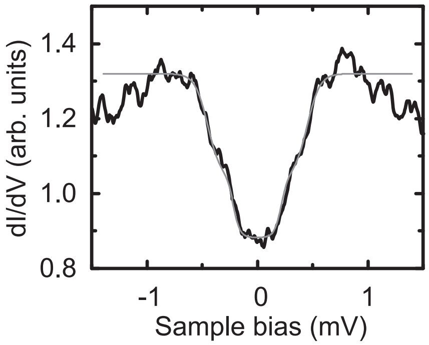

Supplementary Figure 5. MnPc–NH_3 with strong E anisotropy. dI/dV spectrum on an MnPc–NH_3 at $B = 0.1\,T$ acquired with a Au tip. The gap-like feature shows two pairs of symmetric steps around E_F. Setpoint: 5 mV, 200 pA; $U_{mod} = 50\,\mu V_{rms}$. The grey line presents a fit with two pairs of step functions at a sample bias of $\pm(0.22 \pm 0.05)$ mV and $\pm(0.45 \pm 0.05)$ mV, respectively.

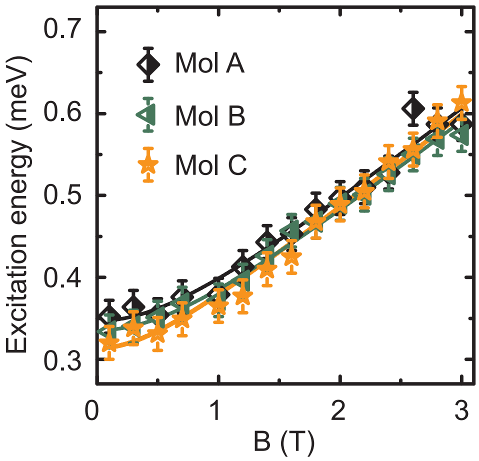

Supplementary Figure 6. B-field dependence of spin excitations. Data points are extracted from least-squares fits with symmetric step functions of the dI/dV spectra measured on three different MnPc–NH_3 complexes A, B, and C at different B-field strengths (B parallel to the sample normal). Error bars account for the standard error of the least-squares fit parameter. The full lines are fits to the Spin Hamiltonian described in the text. Fit parameters in meV: $D_a = -0.24 \pm 0.02$, $E_a = 0.10 \pm 0.02$; $D_b = -0.23 \pm 0.01$, $E_b = 0.11 \pm 0.01$; $D_c = -0.24 \pm 0.01$, $E_c = 0.07 \pm 0.02$.

SUPPLEMENTARY NOTE 1

Controlled NH_3 ligand desorption. The adsorption of the neutral ligand ammonia (NH_3) on MnPc alters the coupling to the Pb substrate without changing the oxidation or spin state as we show in the main text. Supplementary Fig. 1 shows how we can remove the central NH_3 ligand from the molecule in a controlled manner without destroying the order of the molecular island. Supplementary Fig. 1a shows a close-up of a mixed monolayer island of MnPc-NH_3 and MnPc. Next, the STM tip is placed at the position marked by a blue circle and the feedback is disabled. Then, we ramp the bias and record the current (Supplementary Fig. 1c). A sudden drop in the current at $\approx 0.32\,\mathrm{V}$ indicates a change in the junction configuration. The backward sweep (red arrow) confirms an irreversible modification. Scanning the same area again (Supplementary Fig. 1b) then shows the removal of NH_3 from the target molecule and an otherwise unchanged molecular island. Spectroscopic characterization of the molecule after NH_3 desorption shows all features characteristic for MnPc (see below).

SUPPLEMENTARY NOTE 2

Energy of YSR states in MnPc and MnPc–NH_3. Supplementary Fig. 2 shows an island of MnPc–NH_3 and MnPc before (a) and after (b) desorption of NH_3 ligands. To unveil the effect of the axial ligand on the exchange coupling strength between the molecules and the superconductor, we perform dI/dV spectroscopy with a superconducting tip before and after desorption of the NH_3 ligand. Supplementary Fig. 2c (black curves) shows characteristic examples of dI/dV spectra on three different MnPc–NH_3 molecules. For all complexes, we observe a single pair of YSR excitations. While the excitation energy varies from molecule to molecule, the electron-like excitation, i.e., the resonance at positive energy, is more intense for all MnPc–NH_3 molecules studied. This is in contrast to dI/dV spectra on pristine MnPc, which were acquired after the controlled desorption of the NH_3 ligand (pink curves in Supplementary Fig. 2c). The YSR resonances of MnPc are split due to magnetocrystalline anisotropy [1] and show stronger variations in energy and even a shift of the main spectral weight to the negative bias side, i.e., from the electron-like to the hole-like excitations.

For pristine MnPc, the variations of the YSR binding energy were interpreted in terms of a long-range Moiré pattern caused by the incommensurability of the fourfold symmetric molecular island structure with the threefold symmetric substrate. This leads to variations in the adsorption site within the molecular island and, hence, in the exchange coupling strength. As a result, a quantum phase transition of the many-body ground state from a Kondo-screened to a free-spin ground state as indicated by the shift of the spectral weight of the YSR resonances from the positive to the negative bias side is observed [1, 2].

To gain a systematic understanding of the changes induced by the NH_3 ligand on the MnPc, we plot the dI/dV spectra of 44 MnPc molecules with and without NH_3 ligand in pseudo-2D color plots in Supplementary Fig. 2d and 2e, respectively. The spectra are ordered according to the YSR binding energy of the MnPc molecules without ligand. We order the spectra with decreasing energy, i.e., with increasing exchange coupling strength from top to bottom. The spectra of the same molecules but with NH_3 ligand, which were acquired before the ligand was detached via a voltage pulse, are plotted in the same order, i.e., according to the energy of the MnPc YSR states. All YSR binding energies are shifted to more positive values (into a range not observed for MnPc). Interestingly, the order of increasing coupling strength from top to bottom is preserved. Hence, the NH_3 ligand decreased the coupling to the substrate via the surface trans-effect, but does not bleach the differences in the coupling to the substrate induced by the adsorption site differences.

SUPPLEMENTARY NOTE 3

Evolution of the zero-energy resonance with magnetic field. In Fig. 3b of the main text, we present the evolution of the symmetric steps around E_F in a magnetic field perpendicular to the sample surface. For completeness, Supplementary Fig. 3 presents dI/dV spectra at the same B-fields acquired on the same molecule with the same tip in a wider energy range focusing on the zero-energy resonance. While we observe that the gap-like feature at E_F gets more pronounced (this is because the inelastic excitation steps move to higher energy), the zero-energy resonance appears unchanged.

SUPPLEMENTARY NOTE 4

Temperature evolution of the zero-energy resonance - fit to the strong-coupling Kondo model. In the main text, we show that the zero-energy resonance is well described by perturbation theory accounting for third order spin scattering processes. For comparison, we show in Supplementary Fig. 4a fits of the temperature-dependent dI/dV spectra with a Fano-Frota function accounting for temperature broadening. This function describes the transmission for a strong-coupling Kondo impurity, i.e., for $T_\mathrm{K} \gg T$ (T_K is the Kondo temperature, T the experimental temperature) [3–5]. We extract the half width at half maximum (HWHM) from the fits and plot it as a function of T (Supplementary Fig. 4b). A fit to HWHM $= \frac{1}{2}\sqrt{(\alpha k_\mathrm{B}T)^2 + (2k_\mathrm{B}T_\mathrm{K})^2}$ is shown in red (k_B is the Boltzmann constant). According to Fermi liquid theory, in the strong-coupling Kondo regime $\alpha = 2\pi$ [6]. Yet, the least-squares fit yields $\alpha = (3.7 \pm 0.2)\pi$. This clear deviation is another indication for the system to be in the weak-coupling Kondo regime, which renders the determination of a Kondo temperature via a fit of the spectra erroneous [7]. Our interpretation agrees with the zero-field splitting of the resonance (see main text) and is in line with the observed YSR energies of $\epsilon/\Delta \geq +0.5$, which indicate Kondo temperatures well below Δ [8].

SUPPLEMENTARY NOTE 5

Inelastic excitations with two pairs of steps. For most of the MnPc–NH_3 molecules, we observe a single pair of symmetric steps around E_F, which are identified as inelastic spin excitations within the zero-field split spin $S = 1$ manifold. Here, we observe two inelastic excitations which are due to a sizeable rhombicity ($E \neq 0$) and, hence, a lifting of the $M_s = \pm 1$ degeneracy. The fit with two pairs of step functions symmetric to zero yields $D = (-0.34 \pm 0.07)$ meV and $E = (0.11 \pm 0.03)$ meV. Because of the limited energy resolution with a metallic tip ($\approx 300\,\mu$eV at 1.1 K), two pairs of steps are only observed for the largest E anisotropies of the ensemble.

SUPPLEMENTARY NOTE 6

Zeeman shift of inelastic excitations. In Fig. 3e of the main text, we show the B field dependent energies of the inelastic spin excitation of an MnPc–NH_3 complex with vanishing

rhombicity ($E \approx 0$). A linear evolution with B is observed. Here, we present the data of three different molecules with non-vanishing E. At fields larger than 1 T, the excitation energy also increases linearly with field, but below 1 T, the increase is sublinear. This is in line with an easy-axis anisotropy ($D < 0$) with some in-plane distortion ($E \neq 0$).

We fit the data points to a Spin Hamiltonian that assumes the main anisotropy axis to be parallel to the out-of-plane field and accounts for the in-plane distortion:

$$\hat{\mathcal{H}} = DS_z^2 + E(S_x^2 - S_y^2) - g\mu_{\mathrm{B}}B_zS_z. \tag{1}$$

Here, D, E, S_i, B_i , μ_{B}, and g are the axial anisotropy parameter, the in-plane distortion, the projection of the spin and the magnetic field in direction i, the Bohr magneton, and the Landé g-factor, respectively. In order to keep the number of free fit parameters small we restrict g to 2.

SUPPLEMENTARY REFERENCES

[1] Hatter, N., Heinrich, B. W., Ruby, M., Pascual, J. I. & Franke, K. J. Magnetic anisotropy in Shiba bound states across a quantum phase transition. *Nat. Commun.* **6**, 8988 (2015).

[2] Franke, K. J., Schulze, G. & Pascual, J. I. Competition of superconducting phenomena and Kondo screening at the nanoscale. *Science* **332**, 940–945 (2011).

[3] Frota, H. O. Shape of the Kondo resonance. *Phys. Rev. B* **45**, 1096–1099 (1992).

[4] Prüser, H. *et al.* Long-range Kondo signature of a single magnetic impurity. *Nat. Phys.* **7**, 203–206 (2011).

[5] Frank, S. & Jacob, D. Orbital signatures of Fano-Kondo line shapes in STM adatom spectroscopy. *Phys. Rev. B* **92**, 235127 (2015).

[6] Nagaoka, K., Jamneala, T., Grobis, M., and Crommie, M. F. Temperature Dependence of a Single Kondo Impurity. *Phys. Rev. Lett.* **88**, 077205 (2002).

[7] Zhang, Y.-H. *et al.* Temperature and magnetic field dependence of a Kondo system in the weak coupling regime. *Nat. Commun.* **4**, 2110 (2013).

[8] Satori, K., Shiba, H., Sakai, O. & Shimizu, Y. Numerical renormalization group study of magnetic impurities in superconductors. *J. Phys. Soc. Jpn.* **61**, 3239–3254 (1992).
